# An innovative ovine model of severe cardiopulmonary failure supported by veno-arterial extracorporeal membrane oxygenation

**DOI:** 10.1038/s41598-021-00087-y

**Published:** 2021-10-14

**Authors:** Silver Heinsar, Jae-Seung Jung, Sebastiano Maria Colombo, Sacha Rozencwajg, Karin Wildi, Kei Sato, Carmen Ainola, Xiaomeng Wang, Gabriella Abbate, Noriko Sato, Wayne Bruce Dyer, Samantha Annie Livingstone, Leticia Pretti Pimenta, Nicole Bartnikowski, Mahe Jeannine Patricia Bouquet, Margaret Passmore, Bruno Vidal, Chiara Palmieri, Janice D. Reid, Haris M. Haqqani, Daniel McGuire, Emily Susan Wilson, Indrek Rätsep, Roberto Lorusso, Jacky Y. Suen, Gianluigi Li Bassi, John F. Fraser

**Affiliations:** 1 Critical Care Research Group, The Prince Charles Hospital, Chermside, Brisbane, QLD Australia; 2 Faculty of Medicine, University of Queensland, Brisbane, QLD Australia; 3 Intensive Care Unit, St Andrews War Memorial Hospital, Brisbane, QLD Australia; 4 Department of Intensive Care, North Estonia Medical Centre, Tallinn, Estonia; 5 Department of Thoracic and Cardiovascular Surgery, College of Medicine, Korea University, Seoul, Republic of Korea; 6 Department of Pathophysiology and Transplantation, University of Milan, Milan, Italy; 7 Medical ICU, Pitié-Salpêtrière University Hospital, INSERM UMRS-1166, Sorbonne Université, Assistance Publique des Hôpitaux de Paris (AP-HP), Paris, France; 8 Research and Development, Australian Red Cross Lifeblood, Sydney, Australia; 9 Faculty of Medicine and Health, University of Sydney, Sydney, Australia; 10 Science and Engineering Faculty, Queensland University of Technology, Brisbane, QLD Australia; 11 School of Veterinary Science, The University of Queensland, Gatton, Australia; 12 Cardio-Thoracic Surgery Department, Maastricht University Medical Centre, Maastricht, The Netherlands; 13 Institut d’Investigacions Biomèdiques August Pi i Sunyer (IDIBAPS), Barcelona, Spain; 14 Wesley Medical Research, Brisbane, QLD Australia

**Keywords:** Cardiac device therapy, Heart failure, Experimental models of disease

## Abstract

Refractory cardiogenic shock (CS) often requires veno-arterial extracorporeal membrane oxygenation (VA-ECMO) to sustain end-organ perfusion. Current animal models result in heterogenous cardiac injury and frequent episodes of refractory ventricular fibrillation. Thus, we aimed to develop an innovative, clinically relevant, and titratable model of severe cardiopulmonary failure. Six sheep (60 ± 6 kg) were anaesthetized and mechanically ventilated. VA-ECMO was commenced and CS was induced through intramyocardial injections of ethanol. Then, hypoxemic/hypercapnic pulmonary failure was achieved, through substantial decrease in ventilatory support. Echocardiography was used to compute left ventricular fractional area change (LVFAC) and cardiac Troponin I (cTnI) was quantified. After 5 h, the animals were euthanised and the heart was retrieved for histological evaluations. Ethanol (58 ± 23 mL) successfully induced CS in all animals. cTnI levels increased near 5000-fold. CS was confirmed by a drop in systolic blood pressure to 67 ± 14 mmHg, while lactate increased to 4.7 ± 0.9 mmol/L and LVFAC decreased to 16 ± 7%. Myocardial samples corroborated extensive cellular necrosis and inflammatory infiltrates. In conclusion, we present an innovative ovine model of severe cardiopulmonary failure in animals on VA-ECMO. This model could be essential to further characterize CS and develop future treatments.

## Introduction

Cardiogenic shock (CS) is defined as a critical reduction in cardiac output (CO) resulting in end-organ hypoperfusion^1^. Up to 80% of patients with CS develop concomitant acute respiratory failure and require ventilatory support^2–4^. CS can be caused by multiple aetiologies, with acute myocardial infarction (AMI) being the primary cause. Despite improvement in management and advances in therapeutic options, CS is still associated with a mortality of approximately 50% and poor outcomes^5^. Management of CS patients includes treatment of the cause (e.g. coronary reperfusion in AMI) and restoration of adequate end-organ perfusion. The latter can be achieved with medications, ie vasopressors and inotropes, mechanical circulatory support (MCS), or both. MCS use has increased exponentially over the last decade as indications have broadened and early implementation has been shown to improve survival^6–8^. Several MCS systems are used to treat CS, ranging from percutaneous mini-invasive devices—intra-aortic balloon pump (IABP) or micro-axial pumps—to more invasive techniques such as veno-arterial extracorporeal membrane oxygenation (VA-ECMO) and TandemHeart^9^. Among these devices, ECMO use has increased dramatically due to improved management, reduced complications, easier percutaneous access, and improvements in regional planning^8,10^.

The rapid surge in the use of ECMO over the last decade has also highlighted potential adverse effects of ECMO on the heart and other organs (so-called “ECMO—native organ crosstalk”)^11,12^. For instance, the impact on left ventricular (LV) distension and overload, the consequences of differential hypoxia on the brain and coronary arteries and the effect of lower body hyperoxia^13,14^. Considerable progress in improving ECMO has been made, due to the use of large mammalian models of CS and comprehensive experimentation aimed at clinical translation. Indeed, due to the high level of similarities to human in major determinants of myocardial work and energy consumption, e.g. heart rate and vascular wall to-lumen-ratios, large animals remain the method of choice for clinical translation in this field^15^. Nevertheless, the development of a reliable and reproducible large animal model of CS supported by VA-ECMO with significant clinical translatability has been challenging. In particular, reproducing a stable cardiogenic shock model by ligating major coronary arteries often resulted in refractory and life-threatening ventricular arrhythmias^16–19^. Other methods, such as carbon monoxide poisoning or drug-induced CS (beta-blockers), have resulted in heterogeneous outcomes and cause a global decrease in ventricular contractility, different to regional wall motion abnormalities usually seen in myocardial infarction^20,21^.

Thus, the primary aim of this study was to characterise a novel ovine model of CS and pulmonary failure supported by VA-ECMO. In particular, we aimed to develop a model with high level of reproducibility, and reduced risk of refractory arrhythmias that could be reliably applied to assess novel therapeutic strategies and devices that could improve patients outcomes.

## Methods

We present our study methods and results in accordance with the ARRIVE Guidelines for reporting  in vivo research and according to recommendations provided by the European Extracorporeal Life Support Organisation Innovations Workgroup^22,23^.

### Ethics

This study was approved by the University Animal Ethics Committee (UAEC) of Queensland University of Technology (QUT) (Approval no. 1800000337) and adhered to the Australian Code of Practice for the Care and Use of Animals for Scientific Purposes of the National Health and Medical Research Council (NHMRC)^24^. Sheep were managed according to protocols approved by the UAEC.

### Settings

Animal experiments were carried out at the QUT Medical Engineering Research Facility (MERF). Sheep were sourced from the Warwick Saleyards, transported to MERF, and agisted for 2 weeks prior to the beginning of each experiment. During the acclimatization period, sheep had access to food/water ad libitum until the night prior to study commencement, when they fasted. They were regularly examined by MERF veterinarians, and blood tests were performed to confirm the health of the animal (data not provided) prior to experimental procedures. In addition, during the acclimatization period, an echocardiography was performed to assess for normal heart and valvular functions.

### Animal preparation

Six female Merino Cross Dorset non-pregnant sheep (baseline body temperature 39.0 ± 0.5 °C) between 1 and 3 years-old weighing 60 ± 6 kg were used for the study. On the day of the experiment, sheep were transferred to the operative theatre through a custom-made restraining trolley (see Additional File 1, Figure S1). A 7-Fr multi-lumen central venous catheter (Arrow International, USA) was inserted into the left external jugular vein (EJV) under local anaesthesia (5 mL of lidocaine 1%), 1 g of cefazolin was given intravenously for prophylaxis, and animals were pre-medicated with midazolam (0.3 mg/kg). Then, two 8-Fr sheaths (Terumo, Vietnam) were inserted into the left and right EJV under local anaesthesia. Animals were then induced with propofol (3 mg/kg), intubated with a 9.0 endotracheal tube (Smiths Medical, Australia), placed onto the operating theatre in a supine position, connected to a ventilator (Hamilton G5, Hamilton Medical, Switzerland) and monitored non-invasively through pulse oximetry, electrocardiography and capnometry.

#### Initial settings: mechanical ventilation, anaesthesia and monitoring

Mechanical ventilation was initiated using the following settings: tidal volume (VT) of 8 mL/kg, positive end-expiratory pressure (PEEP) of 5 cmH_2_O, respiratory rate (RR) of 15 per minute and inspiratory fraction of oxygen (FiO_2_) of 40%. Settings were adjusted to maintain an arterial partial pressure of oxygen (PaO_2_) between 60 and 100 mmHg and an arterial partial pressure of carbon dioxide (PaCO_2_) between 35 and 45 mmHg. Maintenance of anaesthesia was provided by continuous infusion of midazolam (0.2 to 0.7 mg/kg/h), fentanyl (0.2 to 0.7 mcg/kg/h) and ketamine (0.3 to 0.6 mg/kg/h). Depth of anaesthesia and analgesia was regularly assessed, and medications were adjusted accordingly. A 14-Fr nasogastric tube was gently advanced through the nose and gastric residuals were continuously suctioned throughout the experiment. A 7.5-Fr Swan-Ganz catheter (Continuous Cardiac Output VIP Pulmonary Artery Catheter, Edwards Life Science, CA, USA) was inserted into the left vascular sheath. Finally, 3-Fr arterial cannulas (Yugon, France) were inserted under ultrasound guidance using Seldinger technique via a soft-tip guidewire (Guidewire, 0.018, CR BARD, Covington, GA, USA) into the left and right radial arteries (see Additional File 1, Figure S2). Arterial lines were used for arterial blood sampling and invasive hemodynamic monitoring throughout the experiment.

#### ECMO cannulation

The ECMO circuit comprised a Rotaflow console, centrifugal pump, tubing and a low resistance oxygenator (Quadrox D, Maquet Cardiopulmonary GmbH, Germany). The circuit was primed using 0.9% saline and heated at 37 °C via a heater-cooler machine (Getinge AB, Sweden). Animal was placed in the supine position for cannulation (see Additional File 1, Figure S3). Prior to surgical procedures, vecuronium (0.2 mg/kg) was administered to achieve muscle paralysis. The left femoral artery was exposed surgically through a cut-down technique (see Additional File 1, Figure S4). After 1 h of stabilization, we administered a bolus of heparin (100 units per kg) and corroborated the achievement of activated clotting time ≥ 200 s. Then, a guidewire was inserted into the right external jugular vein sheath and after removal of the sheath, a venous multi-stage 19-Fr cannula (Maquet Cardiopulmonary GmbH, Germany) was advanced down to the inferior vena cava under fluoroscopic guidance. Finally, a 15-Fr arterial cannula (Maquet Cardiopulmonary GmbH, Germany) was inserted through the exposed left femoral artery and advanced up to the descending aorta. After cannulation, ECMO pump speed was increased to 1500 RPM prior to clamp release and was set as follows: pump speed adjusted to achieve a flow rate of 1 L/min, fresh gas flow set at 2 L/min and blender set to deliver 100% oxygen at all times. Blood was then heated to 37 °C. A transfusion threshold for haematocrit and haemoglobin were set as 0.35 and 70 g/L, respectively. Central venous pressure < 4 mmHg or pre-oxygenator pressure < − 50 mmHg were used as criteria to guide vascular filling with 250 mL crystalloid boluses, in order to avoid reduced venous inflow.

### Intervention: cardiogenic shock and acute respiratory failure

#### Development of cardiogenic shock

**Figure 1:**
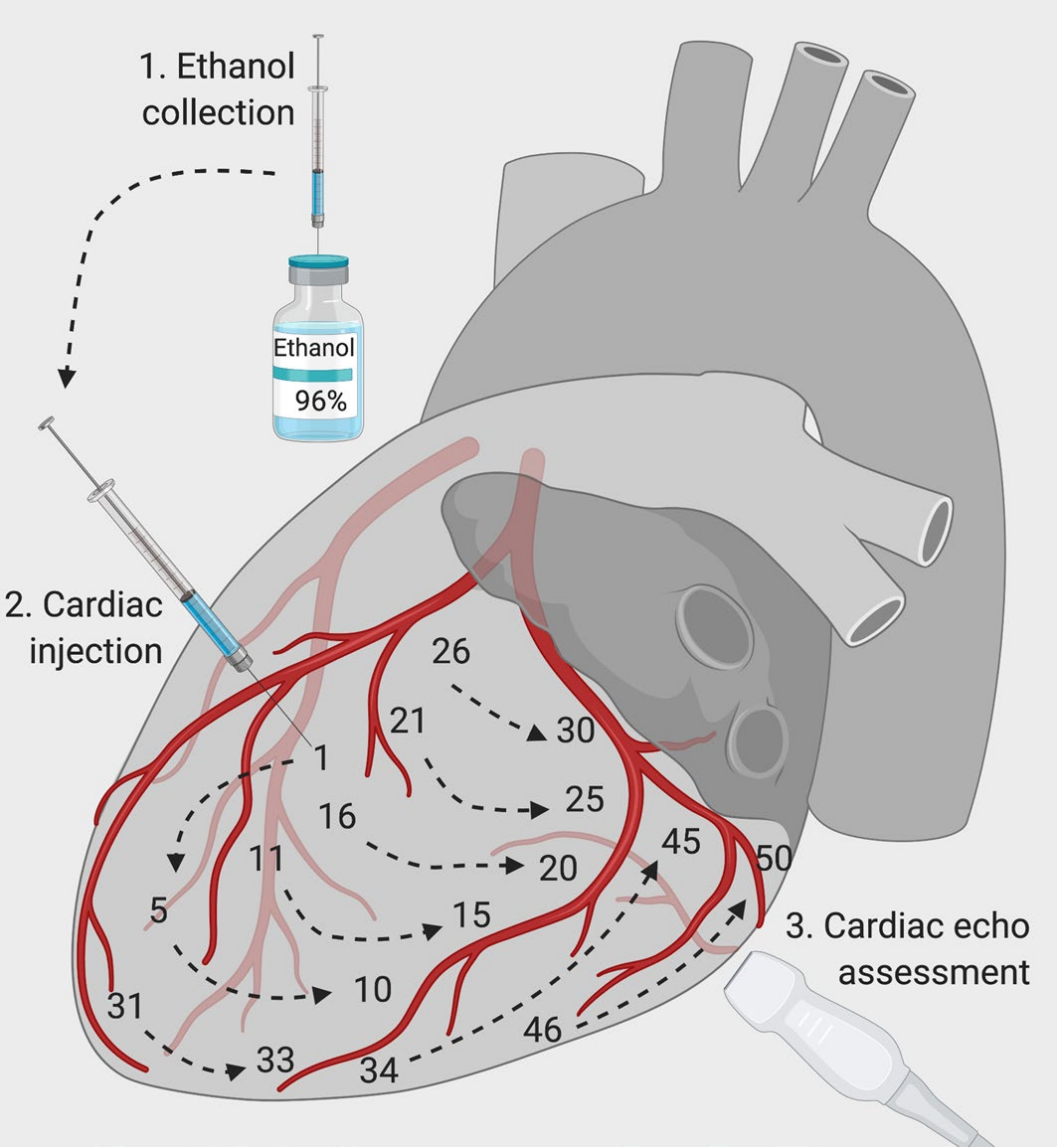
Heart failure induction protocol. Ethanol was injected sequentially with numbers indicating the order of injections (intracoronary injections were avoided). Visual echocardiography was performed to assess the severity of myocardial dysfunction.

Amiodarone (6 mg/kg) and lidocaine (8 mg/kg) were administered prophylactically to avoid ventricular arrhythmias during the procedure. A left lateral mini-thoracotomy was performed at the 5^th^–6^th^ intercostal space to expose the heart. Multiple injections of 96% ethanol were carried out into the subepicardial layer of the anterior, inferior and lateral left ventricle (LV) free wall, parallel and superficial to the LV surface. To avoid intravascular and/or intracavity injections, ethanol was administered only during needle pullback with a 1 mL syringe and a hypodermic 29-G needle or 32-mm 23-G needles for the septum. Additional Movie File shows this in more detail (see Additional File 2). Figure 1 outlines our pre-defined injection protocol. Throughout the procedure, VA-ECMO flow rate was adjusted to ensure mean arterial pressure (MAP) over 65 mmHg, as the LV function progressively weakened. Global and regional contractility was sequentially assessed after 30 injections, and every 10 injections thereafter, through direct epicardial echocardiography. In addition, after visual echocardiography assessment, we reduced ECMO blood flow rate to 1 L/min, and an arterial blood gas (ABG) analysis was carried out to evaluate if CS criteria^1^, as listed below, were met to cease ethanol injections:

- Left ventricle ejection fraction (EF) below 30%;
- Reduction of arterial systolic blood pressure below 90 mmHg for more than 10 min;
- Arterial blood lactate > 4 mmol/L;
- Urinary output < 0.5 mL/kg/h.

#### Development of acute respiratory failure

After the development of CS, we allowed a 1 h of stabilization, ventilatory support was then substantially decreased to develop acute respiratory failure (ARF): VT was reduced to 4 mL/kg, PEEP was reduced to 0 cmH_2_O, RR was set to 5/min and FiO_2_ was set at 21%. Thus, after 1 h from ventilatory adjustment, ARF was confirmed through the withdrawal of blood from the right radial artery (expected VA-ECMO mixing zone from the distal aortic arch to descending aorta), indicating PaO_2_ below 60 mmHg.

### Experimental protocol and assessment

#### Blood sampling

**Figure 2:**
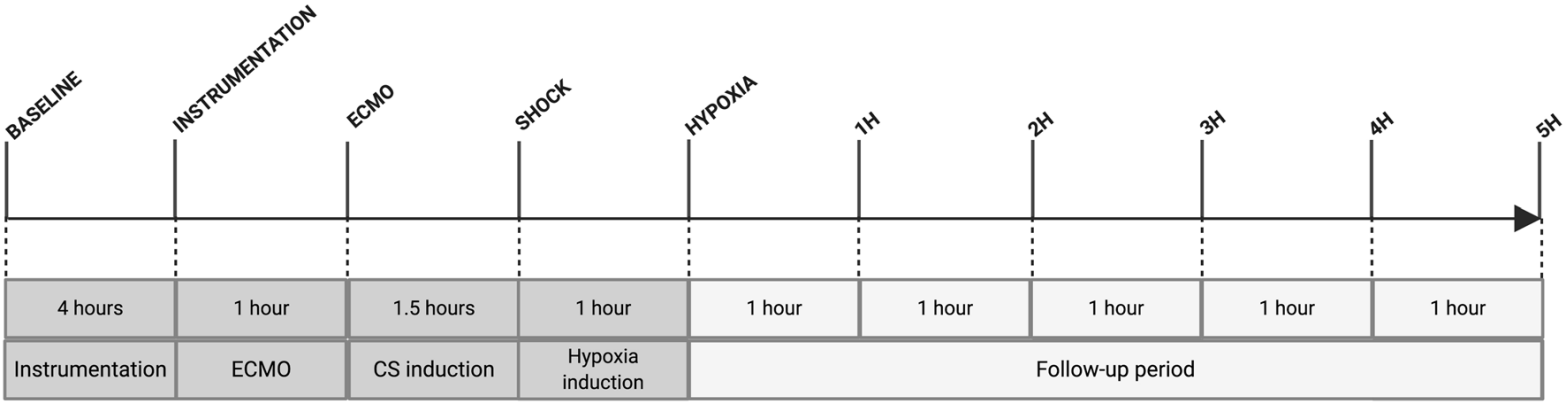
Experimental timeline. Approximate length of the experiment was 12 h. *CS* cardiogenic shock, *ECMO* extracorporeal membrane oxygenation.

Arterial blood samples from the right radial artery were collected at 1-h post-instrumentation, upon ECMO cannulation, CS induction (T_shock_), ARF development (T_hypoxia_) and hourly during the 5-h follow-up period (Fig. 2). Severity of heart injury was further corroborated by cardiac Troponin-I (cTnI) assay (Cat#CTNI-9-HSP, Fisher Biotech, Australia).

### Cardiac evaluation

Full methodology entailing echocardiography and electroanatomical mapping is outlined in Additional File 1. In brief, epicardial echocardiography was performed at the time points reported in Fig. 2. Conventional parasternal short axis views were obtained. We assessed left ventricular end-diastolic area (EDA), left ventricular end-systolic area (ESA), fractional area change (FAC), endocardial global circumferential strain (GCS) and global radial strain (GRS).

### End of the study and post-mortem analysis

At the end of the 5-h follow-up, animals were euthanised and the heart was taken for histological assessment. Tissue was fixed in 10% neutral buffered formalin for at least 24 h, embedded in paraffin, sectioned to a 5 µm thickness and stained with hematoxylin and eosin (H&E) using standard procedures. Slides were examined by an independent, blinded, specialist veterinary pathologist and scored for features listed in Table 2.

### Statistical analysis

Data are presented as mean ± standard error of the mean (SEM) or median with interquartile range (IQR) when appropriate. A dependent t-test was fitted to determine the effect of cardiorespiratory failure induction in the animals. A repeated measures one-way ANOVA was used to analyse dynamic changes in cTnI levels and echocardiographic parameters. To assess the impact of number of injections on key parameters of cardiac failure we carried out a restricted maximum likelihood analysis, based on repeated measures approach, including number of injection and times of assessment upon and after development of cardiac failure (Shock, Hypoxia, 1H, 2H, 3H, 4H and 5H). A (co)variance structure was used to model the within-subject errors and the Kenward-Roger approximations to estimate denominator degrees of freedom. For each continuous variable, the overall F test was first assessed for significance (p ≤ 0.05). If it was significant, all two-sided comparisons among groups were performed. Each pair-wise comparison was corrected using Bonferroni test, to control for the experiment-wise error rate. Normality of the residuals of the mixed models were assessed to confirm reliability of the model. Statistical analyses were performed using R statistical software package version 4.0.2 (2020-06-22) (R Core Team 2020).

## Results

### Population

All six animals survived (100%) until the end of the study and were included in the final analysis. In addition, baseline body temperature and haemoglobin were 39.0 ± 0.5 °C and 10.9 ± 1.4 g/dL, respectively.

### Development of CS and ARF

A median number of 53 injections (IQR 40–72, minimum 36, maximum 100) of 96% ethanol was administered to the animals to achieve CS. An associative trend between number of injections and global circumferential strain was found (p = 0.051) on linear mixed model analysis, while number of injections did not impact lactate (p = 0.612), global radial strain (p = 0.249) or FAC (p = 0.139). Table 1 depicts key parameters at baseline and upon CS development. Systolic arterial blood pressure dropped by 30.9% (p = 0.009), while lactate increased by 29.8% (p < 0.001). Importantly, FAC decreased from 34 ± 9 to 16 ± 7% (p = 0.003). The reduction in respiratory support resulted in a statistically significant drop of PaO_2_ from 98.3 ± 36.2 mmHg to 45.5 ± 12.6 mmHg (p = 0.01) and pH (7.38 ± 0.02 *versus* 7.25 ± 0.08, p = 0.01), but no significant change in PaCO_2_ (47 ± 3 mmHg *versus* 45 ± 10 mmHg, p = 0.73).

**Table 1:** Haemodynamic parameters and cardiac assessment pre- and post-induction of cardiogenic shock.

	Baseline	CS	p-value
**Haemodynamic parameters**			
Heart rate (BPM)	85 ± 18	99 ± 17	0.06
**SAP (mmHg)**	**97 ± 18**	**67 ± 14**	**0.01**
MAP (mmHg)	80 ± 18	56 ± 11	0.03
CO^a^ (L/min)	4.1 ± 0.8	–	–
SV^a^ (mL)	46 ± 13	–	–
SVRI^a^ (dynes/cm^5^/m^2^)	1697 ± 646	–	–
**Lactate (mmol/L)**	**1.4 ± 0.8**	**4.7 ± 0.9**	** < 0.001**
**Diuresis (mL/kg/h)**	**2.1 ± 1.6**	**0.7 ± 0.8**	**0.09**
**Cardiac assessment**			
Fractional area change (%)	34 ± 9	16 ± 7	0.003
**LVEF (%)**	**42–52**	**15–29**	–

Parameters in bold are part of the definition of CS according to the latest guidelines^1^. Data presented as mean ± SD.

*BPM* beats per minute, *CO* cardiac output, *LVEF* left ventricular ejection fraction, *MAP* mean arterial pressure, *SAP* systolic arterial blood pressure, *SV* stroke volume, *SVRI* systemic vascular resistance index.

^a^CO, SV and SVRI were not measured after commencement of VA-ECMO due to inappropriateness of Swan-Ganz measurements during ECMO.

### Cardiac assessment

**Figure 3:**
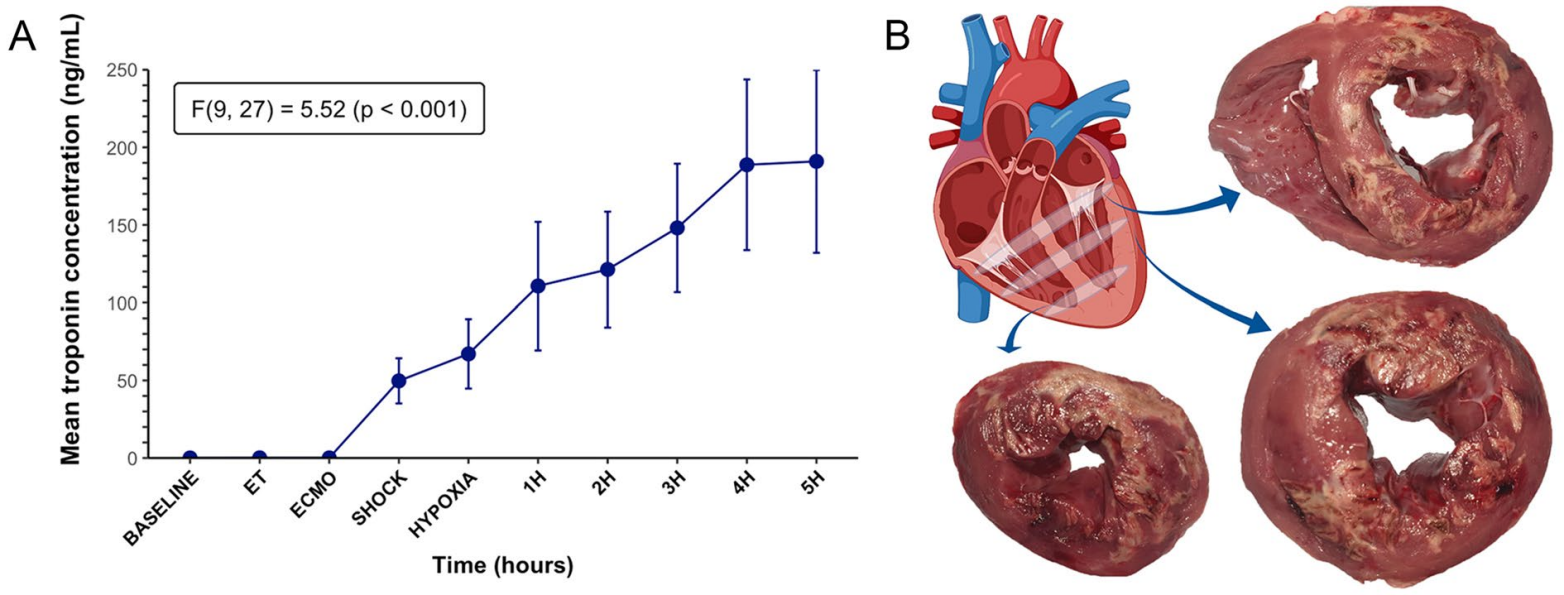
Appraisal of cardiac injury. (**A**) Plasma levels of cardiac Troponin I increased significantly over time from ECMO to 5 h. Data are presented as mean ± SEM, n = 6. (**B**) Macroscopic images of short-axis base, mid and apical sections of the ovine heart obtained after 5 h of follow-up post-cardiogenic shock and acute respiratory failure induction. *ECMO* extracorporeal membrane oxygenation, *ET* end of instrumentation.

Figure 3 outlines the severity of cardiac injury. cTnI increased significantly throughout the study (Fig. 3A, p < 0.001). As seen on segments of cross-sectional heart tissue (Fig. 3B), ethanol injection resulted in areas of transmural necrosis with well-demarcated margins. Necrosis was seen on the anterolateral, posterior, and septal heart wall regions.

### Echocardiography

**Figure 4:**
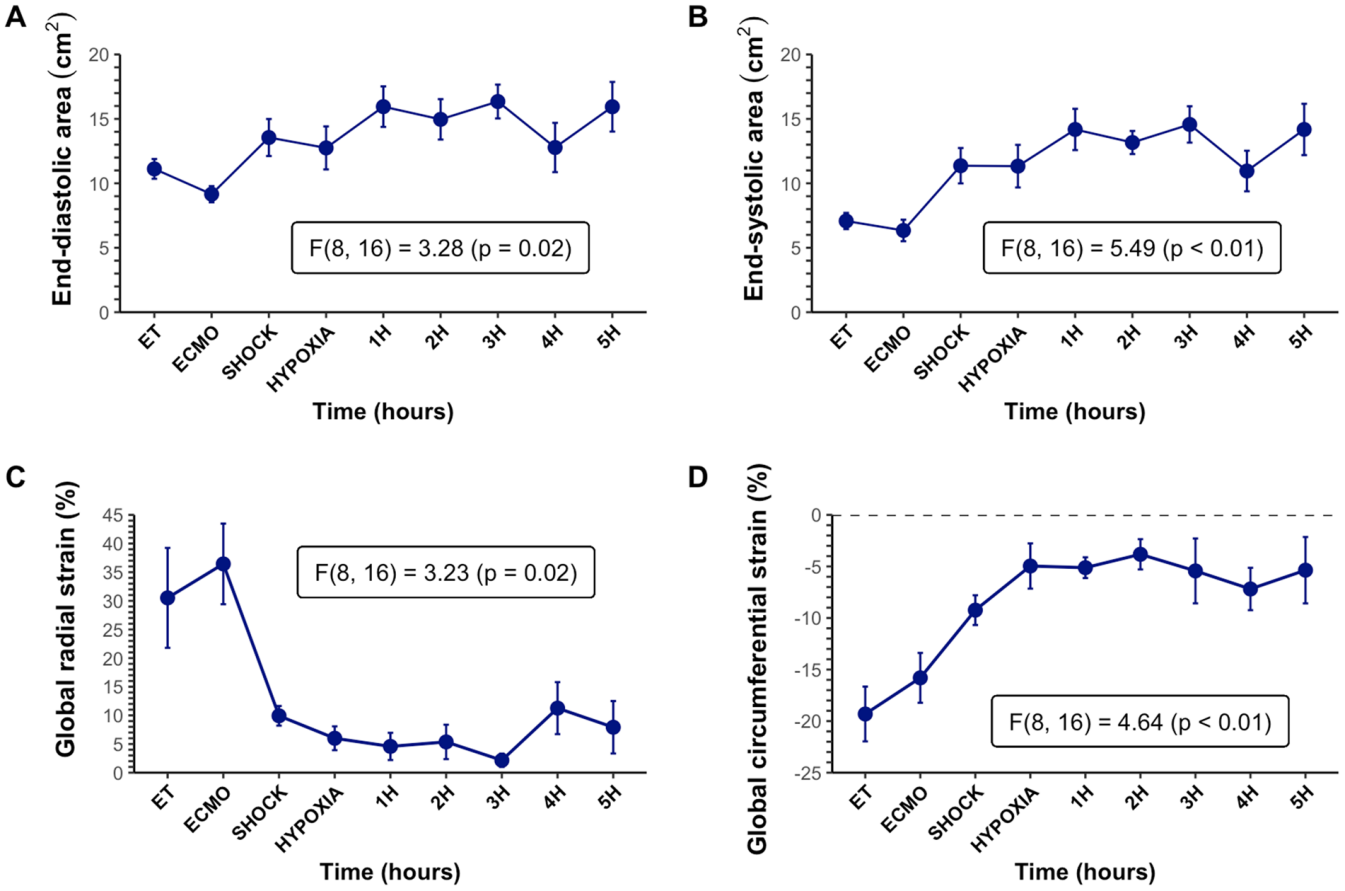
Echocardiographic assessment. (**A**) Left ventricular end-diastolic areas throughout the experiment. There was a statistically significant increase in the end-diastolic area after cardiogenic shock was induced and full-support ECMO was initiated. (**B**) Left ventricular end-systolic areas increased similarly after full-support with ECMO was commenced. (**C**) Global radial strain decreased significantly from 35 to 10% after cardiogenic shock was induced. (**D**) Global circumferential strain showed a decrease to a lesser degree from − 18 to − 9% after shock was induced. *ECMO* extracorporeal membrane oxygenation, *ET* end of instrumentation.

After development of CS, epicardial echocardiography revealed a statistically significant increase in both left ventricular end-diastolic (Fig. 4A) and end-systolic (Fig. 4B) areas. The absolute values of both GRS (Fig. 4C) and GCS (Fig. 4D) decreased significantly after CS and remained low throughout the follow-up phase of this experiment.

### Histology

**Figure 5:**
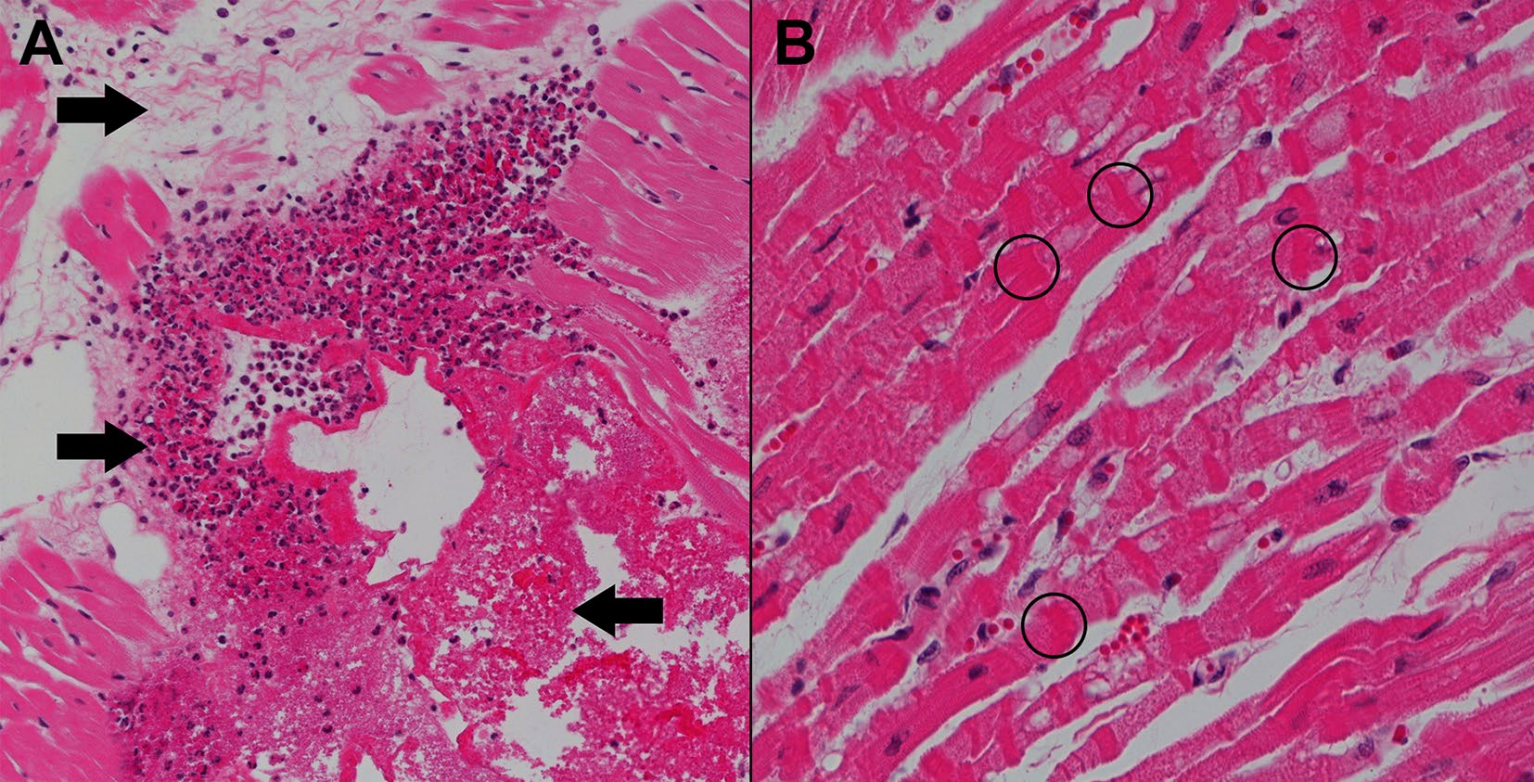
Representative H&E-stained tissue sections demonstrating primary lesions with end-organ injury associated with cardiogenic shock. (**A**) Left ventricle of the heart (×20 magnification), showing necrotic lesions (right arrow), oedema (top left arrow) and inflammatory infiltrates (bottom left arrow). (**B**) Right ventricle of the heart (×40  magnification), showing contraction band necrosis with thick and intensely eosinophilic staining contraction bands (circles).

Cardiac tissue histological assessment of the left ventricle showed substantial injury (Fig. 5A, Table 2), characterized by coagulative necrosis, neutrophilic infiltration and intramuscular haemorrhage. The right ventricle also presented a wide spectrum of changes ranging from contraction band necrosis (Fig. 5B) to myocytolysis and coagulative necrosis, consistent with end-organ damage seen in shock.

**Table 2:** Histopathological scoring system with left-ventricular (primary lesion) and right ventricular (end-organ) tissue injury score.

Organ	Histological feature (magnification)	Grade 0	Grade 1	Grade 2	Mean score
Heart *Left ventricle*	Contraction band necrosis (40×)	None	Single focus	Multiple foci	0.74 ± 0.32
	Neutrophilic infiltration (40×)	None	< 20 neutrophils	> 20 neutrophils	0.94 ± 0.34
	Intramuscular haemorrhage (20×)	None	< 50% affected	> 50% affected	0.94 ± 0.50
	Oedema (interstitial or perivascular) (20×)	None	< 50% affected	> 50% affected	0.5 ± 0.14
	Myocytolysis, necrosis (20×)	Not present or single cell	Groups of cells, focal	Groups of cells, several foci	1.16 ± 0.09
Heart *Right ventricle*	Contraction band necrosis (40×)	None	Single focus	Multiple foci	0.28 ± 0.24
	Neutrophilic infiltration (40×)	None	< 20 neutrophils	> 20 neutrophils	0.25 ± 0.14
	Intramuscular haemorrhage (20×)	None	< 50% affected	> 50% affected	0.22 ± 0.30
	Oedema (interstitial or perivascular) (20×)	None	< 50% affected	> 50% affected	0.1 ± 0.13
	Myocytolysis, necrosis (20×)	Not present or single cell	Groups of cells, focal	Groups of cells, several foci	0.36 ± 0.10

### Electrophysiology

**Figure 6:**
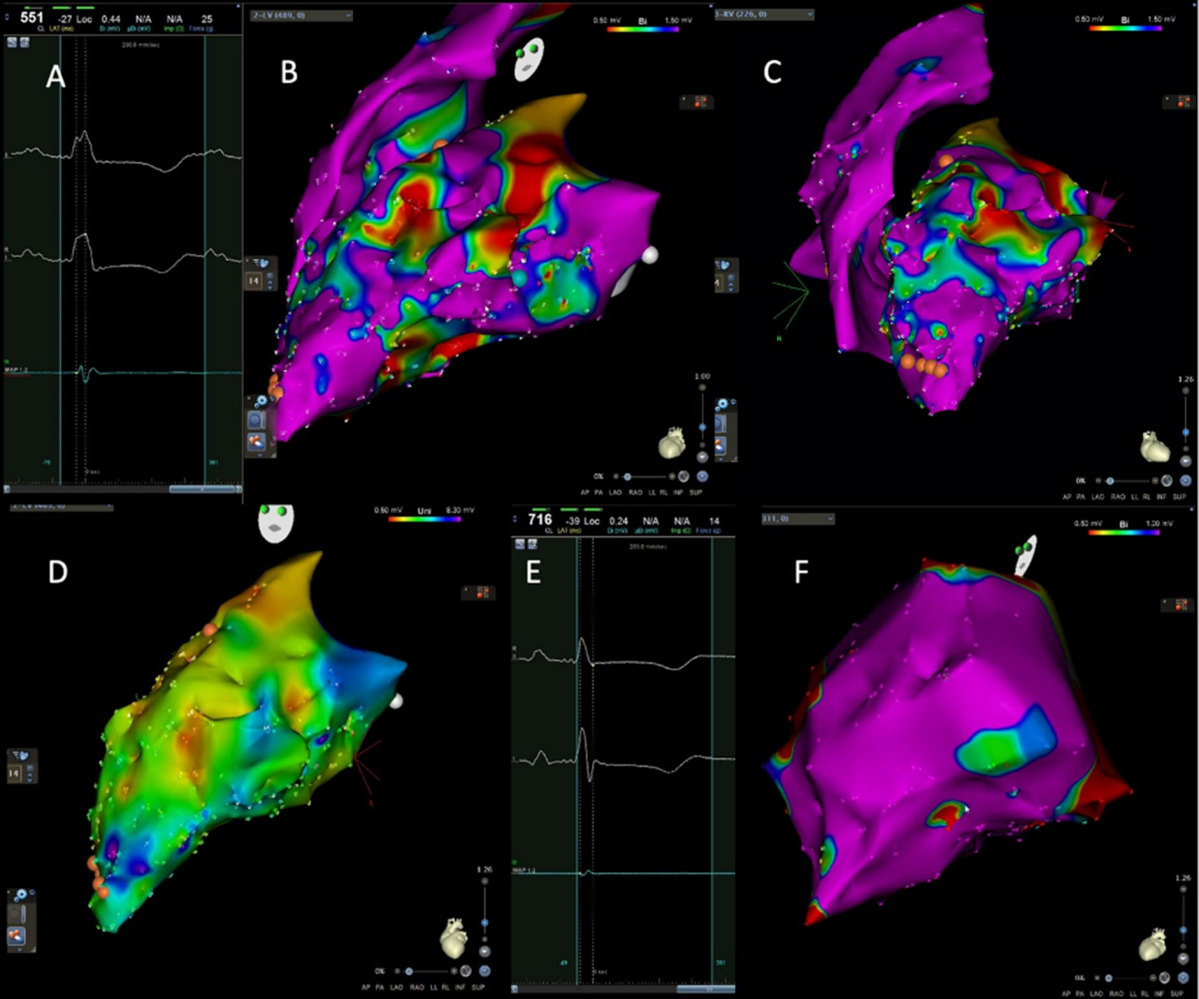
Overview of left and right ventricular cardiac electroanatomic substrate mapping. (**A**) Attenuated bipolar electrograms from the LV endocardium. (**B**) Endocardial biventricular substrate maps in the left lateral projection. (**C**) Endocardial biventricular substrate maps in the LAO projection. B and C show patchy zones of bipolar voltage attenuation with areas in red corresponding to dense scar (voltage < 1.5 mV). (**D**) Significant endocardial LV unipolar voltage attenuation in the LAO projection. (**E**) Abnormal low voltage epicardial. (**F**) LAO caudal projection of an epicardial substrate map showing relative epicardial bipolar voltage preservation. *LV* left ventricle, *LAO* left anterior oblique, *RAO* right anterior oblique, *AP* anteroposterior.

Electroanatomic substrate mapping in one animal showed discrete zones of patchy bipolar and unipolar electrogram voltage attenuation corresponding to the presumed sites of ethanol injection (Fig. 6). Endocardial mapping revealed this in the mid anterolateral and inferior LV, as well as on the septum. In total, 29.4 cm^2^ of the LV endocardial surface was scarred by electrogram criteria, corresponding to 19.7% of the chamber surface area. Conversely, electroanatomic mapping of the RV was unremarkable. Epicardial voltages were relatively preserved compared to the endocardium and showed only patchy scar zones. Left ventricular electrogram morphology showed marked peak-to-peak signal attenuation but no isolated, split, or fractionated electrograms were seen (Fig. 6A,E).

### Complications

Arrhythmic complications occurred in two animals. In one animal, ethanol injection into the septum was complicated by third-degree atrioventricular block with a slow escape at 20 bpm, most likely due to direct damage to the atrioventricular conduction system. A second animal developed ventricular fibrillation 80 min after the development of CS but was successfully resuscitated by epicardial defibrillation (12 shocks in total), and administration of further amiodarone (10 mg/kg) and lidocaine (8.3 mg/kg), while VA-ECMO blood flow rate was temporarily and appropriately increased to 4 L/min.

## Discussion

We present a reproducible ovine model of severe cardiopulmonary failure supported by VA-ECMO. CS was induced by ethanol injection and confirmed by significant changes in pre-defined hemodynamic (SAP < 90 mmHg), metabolic (lactate > 4 mmol/L) and echocardiographic criteria (LVEF < 30%), consistent with CS definitions from contemporary clinical trials and guidelines^1^. These were further corroborated by pathognomonic biochemical, macroscopic, and histopathological features. Acute respiratory failure was induced by lowering mechanical ventilatory support and confirmed by pre-defined hypoxic criteria in ABG analysis.

Heart failure models should be induced to reflect various types of clinical situations, especially for mechanical circulatory support devices (such as ECMO) for profound cardiogenic shock. A systematic review by our group found that 7 out of the 18 studies investigating CS were unsuccessful at meeting a wide range of CS diagnostic criteria and would rather be described as acute heart failure models, rather than cardiogenic shock models^22^. Methodological limitations of large animal trials can risk the clinical translation of their findings, hence particular attention was paid to confirm the extent of shock in all animals^25,26^.

### Confirming site-specific injury: electrophysiology

Ventricular fibrillation is the most frequent side-effect of previous experimental cardiogenic shock models, leading to death in up to 50% of animals^16,17,21,27^. To reduce the loss of animals, antiarrhythmics were administered before ethanol injection, and this resulted in ventricular fibrillation in only one animal. Complete heart block developed in one animal, likely due to severe ethanol-induced injury to the atrio-ventricular conduction system. These serious arrhythmogenic events were limited and did not affect the mortality rate of our animal cohort. To further characterize this model electrophysiologically, both endo- and epicardial electroanatomic substrate mapping were performed in one animal. This showed that almost 20% of the endocardial LV surface had electrograms attenuated to an extent consistent with scar. This attenuation was not confluent but was seen in discrete zones of regional endocardial and epicardial LV myocardium corresponding to injection sites. The electrograms in these zones were attenuated but did not exhibit fractionation or isolated delayed components. This is consistent with the histological demonstration of dense confluent cellular necrosis in the injected regions without intervening surviving myofiber bundles. Epicardial voltages were relatively preserved, consistent with a zone of subepicardial myocyte preservation related to the depth of ethanol injection. Thus, formation of myocardial necrosis is directly related to injury site. Increased number of sites injected relates to expansion of scar area, allowing for titration of left-ventricular failure.

### Advantages of ethanol-based model of CS

As detailed in Additional File 1 (Summary S1), prior to creating this model currently being described, we unsuccessfully attempted to develop CS using an extensive dose of intravenous esmolol. There was substantial variability in cardiac function and pre-determined CS criteria were not met, hence we proceeded with novel methods through ethanol injections. In the model presented, ethanol injection resulted in an isolated and loco-regional left ventricular wall motion abnormality, with changes being limited to injection site. This achieves multiple desirable goals:

1. Injury can be created regionally, and wall motion abnormality can be localized directly,
2. The extent of injury is potentially titratable – researchers can study decreasing levels of heart failure,
3. The pattern of injury can replicate a desired clinical scenario (right heart infarction, anterolateral infarction etc*.*) with high precision in multiple animals.

Direct ethanol injections for the development of right heart failure have been previously demonstrated by Thomaz et al., with isolated right heart failure induced by direct ethanol injection in 13 dogs^28^. This study showed a 74% transmural infarction area, limited to the right ventricle, assessed on the 14th day after injury was induced. To date, only one study by Marques and collaborators has reported the use of intramural left ventricle ethanol injections, unfortunately with limited and inconclusive description of outcomes and no use of VA-ECMO to achieve considerable levels of cardiac failure^29^. Several experimental heart failure models have been published with intracoronary ethanol injections^30,31^. However, the pathogenesis of these models is essentially distinct from an intramural injection model as the former achieves LV injury by promoting a persistent and extensive constitution of thrombi distally to the injection site, while the latter induces myocardial necrosis without intraluminal thrombi^32,33^. Effects of intraluminal injection disperse to the respective coronary vascular bed, while intramural injection limits the injury to its specific site. A site-specific injury pattern achieved with our model enables researchers to create specific clinical scenarios and allows for pre-clinical validation in a randomized setting, ultimately giving clinicians better guidance when it comes to choosing the correct treatment for the right patient.

### Novel animal model of CS within the context of previously developed models of CS

Our model differs from previously published models, in its ability to reproduce a stable and controllable CS.

To date, pre-clinical heart failure models have employed a range of different animals (primarily sheep, pigs, and dogs) and methods (e.g., coronary ligature or occlusion, rapid pacing, myocardial hypoxia)^15^. The majority of patients who develop acute CS requiring MCS have an overwhelming AMI; therefore, unlike chronic heart failure models (e.g., rapid pacing, mitral regurgitation models), experimental acute heart failure models have predominantly been created through coronary artery occlusions (surgical or intravascular). However, the latter can frequently lead to critical and unpredictable adverse events, such as refractory hemodynamic instability caused by ventricular arrhythmias or sudden cardiac arrest^34^. Other models, such as global hypoxia, esmolol-induced LV failure or carbon monoxide poisoning have limited translatability, as they induce global LV failure which is rarely seen in AMI patients. Therefore, in comparison with previous models, our model overcomes limitations of achieving a myocardial injury which is either excessively severe or marginal, without possibility to titrate the degree of injury. Furthermore, we conducted ECMO cannulation prior to shock induction to ensure high survival despite the considerable degree of heart failure.

### Study limitations

Several limitations of our model should be highlighted. Firstly, whilst on peripheral VA-ECMO, ejection fraction decreases as afterload increases. Thus, during ECMO, left ventricular outflow tract velocity time integral could be a reliable estimate of heart function, independently of the afterload. Due to dissimilarities between the human and sheep thorax anatomy, measurement of outflow tract velocity time integral was not obtained. Yet, to minimize the effect of increased afterload, we obtained our echocardiographic assessments at baseline and at CS, using only 1 L/min of VA-ECMO support to minimize afterload, as is often applied in clinical practice to assess the possibility of weaning patients from VA-ECMO^35^. Secondly, only six animals were evaluated, yet consistent results were found amongst all the evaluated animal subjects. Thirdly, to achieve ethanol administration, the thorax was opened, which is known to modify the heart–lung interactions in mechanically ventilated patients^36^. Indeed, opening the thorax will cancel the effect of MV on stressed vascular volume, in particular the increase in RV afterload, decrease in LV afterload and venous return. Nevertheless, an open chest model was essential to titrate and evaluate injury in these animals. In addition, we carried out cardiac mapping in only one animal, due to the extensive costs of these assessments. Although we obtained critical findings, further corroboration of electrophysiological impairments associated with ethanol injections should be obtained in future studies. Finally, although ethanol was injected into the myocardium while slowly pulling out the needle to avoid inoculation into the bloodstream, ultrasound-guided injections could further improve safety of the intervention.

### Prospects for future studies in CS: VA-ECMO and increased left ventricular overload

As previously outlined, although ECMO can provide essential restoration of end-organ perfusion, this technology is not devoid of potential hazards. Of these, it’s increasingly recognized that peripherally instituted VA-ECMO can hamper cardiac recovery as it exerts stress and strain on the left ventricular myocardium^37^. This so-called cardiac overload is clinically relevant, since increased filling pressures can promote pulmonary oedema and formation of intraventricular thrombi with embolic events, which can drastically worsen patient outcomes irrespective of the use of VA-ECMO^38,39^. The effect of increasing VA-ECMO flow on left-ventricular dimensions has been comprehensively assessed by Ostadal et al*.* in a porcine model of cardiogenic shock^18^. The authors reported an increase of left ventricular volumes as ECMO flow was incrementally increased. Similar findings were present in our model: EDA and ESA increased significantly after full-flow ECMO support was initiated. Importantly, use of unloading devices, i.e. Impella is associated with decreased mortality at the cost of increased bleeding rates^40^. Although other unloading methods, such as pharmacological therapies or LV venting exist, increased LV afterload is a crucial clinical complication. Thus, in conjunction with continuous measurement of intracardiac pressures and volumes, our model could be crucial for future studies investigating innovative unloading technologies for VA-ECMO during CS. Furthermore, whilst we recognize this model as a novel tool to investigate device therapies in CS, we must acknowledge its limitations for pharmacological experimentation. CS was achieved through direct toxic myocardial necrosis, which doesn’t reproduce ischemia and reperfusion phases with a penumbra myocardial area and the complex phenomenon of re-entry which triggers frequently lethal ventricular arrhythmias.

## Conclusions

We herein described and comprehensively assessed a novel model of severe cardiogenic shock and ARF achieved by direct intramural injection of ethanol to the left ventricle and marginal support by the mechanical ventilator. Our methods produced a clinically relevant decline in left ventricular performance with a hemodynamic profile consistent with CS. Our model is stable, and injury can be induced in a site-specific manner, making it highly suitable for future studies investigating device therapies in CS which aim to reproduce a specific clinical scenario; and takes critical care (e.g., mechanical circulatory support) closer to personalised medicine.

## Supplementary Information

Supplementary Information 1.

Supplementary Video S1.

## Data Availability

The datasets used and/or analysed during the current study are available from the corresponding author on reasonable request.
