# Supplementary material for: An innovative ovine model of severe cardiopulmonary failure supported by veno-arterial extracorporeal membrane oxygenation
Source: Sci Rep. 2021 Oct 14;11:20458. doi: 10.1038/s41598-021-00087-y (PMC8516938; doi:10.1038/s41598-021-00087-y)
Supplement: Supplementary file 1 — Supplementary Information 1. [file 41598_2021_87_MOESM1_ESM.docx]

**An innovative ovine model of severe cardiopulmonary failure supported by veno-arterial extracorporeal membrane oxygenation**

**Additional File 1, Supplemental Material**

**Summary S1:** Development of cardiogenic shock model through esmolol infusion

**Methods S1**: Electrophysiological assessment and echocardiography

**Figure S1**. Sheep prior to induction and endotracheal intubation restrained into a custom-made trolley.

**Figure S2.** Radial artery cannulation.

**Figure S3**. Animal in supine position after venous-arterial extracorporeal membrane cannulation through the right jugular vein (inflow) and left femoral artery (outflow).

**Figure S4**. Left femoral artery was accessed through cut-down technique prior to cannula insertion.

**Summary S1: Development of cardiogenic shock model through esmolol infusion**

**Methods**

We studied three female Merino Cross Dorset non-pregnant sheep (48 ± 3 kg) that were instrumented as described in the main manuscript. However, no thoracotomy was performed since intravenous infusion of esmolol through the central venous catheter was carried out to impair cardiac function. Thus, after instrumentation and stabilisation, an initial esmolol bolus up to 1 mg/kg was infused over 10 minutes. Then, up to 500 mcg/kg/min of esmolol was infused continuously to achieve cardiogenic shock diagnostic criteria, as described in the main manuscript. Animals were followed up 3 hours, then, euthanasia was performed, and organs were harvested.

**Successful development of cardiogenic shock**

In one out of three sheep, 40 mg (0.89 mg/kg) of esmolol was given as a bolus, which rapidly impaired cardiac function with loss of pulsatile arterial pressure waveform under support of venous-arterial extracorporeal membrane oxygenation (VA-ECMO) at 2.5 L/min of blood flow. Furthermore, systolic blood pressure decreased from 116 mmHg to 75 mmHg, lactate increased from 0.5 mg/L to 3.2 mg/L and urinary output decreased from 2.3 mL/kg/h to 0.68 mL/kg/h.

**Unsuccessful development of cardiogenic shock**

In two out of three animals, maximal doses of esmolol did not result in a consistent and reliable model of cardiogenic shock. In one animal, esmolol (4 mg/kg) bolus did not cause significant cardiac impairment, but led to refractory ventricular fibrillation. Irrespective of the support by VA-ECMO (blood flow up to 4.0 L/min) and epicardial defibrillation (20 shocks, up to 30 Joules) return to spontaneous circulation was never achieved and the experiment was prematurely stopped. In another animal, esmolol infusion was progressively increased from 50 mcg/kg/min up to 1 mg/kg/min, but the animal remained hemodynamically stable. Thus, the study was prematurely stopped for lack of response to esmolol infusion.

**Conclusion**

Due to the high rate of failed attempts (2 out of 3 experiments) in developing a cardiogenic shock model in sheep with the use of esmolol, we decided to change methods and shift to the model of ventricular injury through ethanol injections, as described in the main manuscript.

**Potential limitations of esmolol model of cardiogenic shock**

Esmolol is an ultra-short-acting beta-adrenergic blocking agent, which is rapidly hydrolysed by red blood cell esterases, forming ASL-8123 and methanol inactive metabolites [1]. In a goat model of cardiogenic shock by Naito and colleagues, the investigators injected esmolol directly into the left atrium. This alternative route could have exerted direct myocardial prior to esmolol metabolization [2]. The authors confirmed cardiogenic shock 40% reduction of cardiac output, reduction in aortic pressure greater than 20 mmHg, and increase in left atrium pressure greater than 10 mmHg. In our early experiments, although we injected esmolol through the central venous catheter placed into the left jugular vein, we were unable to reproduce positive results reported by Naito and collaborators. In addition, we did not carry out systematic echocardiographic evaluation of left ventricular function, which is oftentimes performed in clinical settings to corroborate the degree of cardiac depression and aetiology.

**REFERENCES:**

1. Reynolds RD, Gorczynski RJ and Quon CY. Pharmacology and Pharmacokinetics of Esmolol. *The Journal of Clinical Pharmacology*. 1986;26:A3-A14.

2. Naito N, Nishimura T, Iizuka K, Fujii Y, Takewa Y, Umeki A, Ando M, Ono M and Tatsumi E. Novel Rotational Speed Modulation System Used With Venoarterial Extracorporeal Membrane Oxygenation. *Ann Thorac Surg*. 2017;104:1488-1495.

**Methods S1**: Electrophysiological assessment and echocardiography

*Electrophysiology*

In one animal, biventricular endocardial and epicardial electroanatomic substrate mapping was performed after 2 hours from CS and ARF development. For this purpose, an additional 9-Fr vascular sheath was introduced into the right femoral vein. Using the CARTO® 3-dimensional electroanatomic mapping system (Biosense Webster, Diamond Bar, CA, USA), point-by-point mapping was performed using a 3.5 mm tip mapping catheter with a 1 mm tip-to-ring interelectrode distance (Thermocool® Smarttouch®, Biosense Webster, Diamond Bar CA, USA) during sinus rhythm. Geometry and bipolar/unipolar electrograms were simultaneously recorded from the endocardial right and left ventricles and directly from the epicardium, ensuring adequate sampling of all segments. Bipolar signals were recorded between the distal tip and ring electrodes and filtered at 30 to 400 Hz. Unipolar signals were measured between the tip electrode and Wilson’s central terminal and were filtered at 1 to 240 Hz. The fill threshold was set to 15 mm. Electroanatomic scar was defined as a zone of bipolar electrograms with peak-to-peak amplitude of <1.5mV.

*Echocardiography*

Epicardial echocardiography (IE-33, Philips, North Ryde, Australia, or GE Vingmed Ultrasound, Horten, Norway) was performed at the time points reported in **Figure 2**. Conventional parasternal short axis (PSAX) views, using ECG gating, were obtained. All echocardiographic images were transferred to a separate workstation and analysed using dedicated software (TomTec-Arena, TomTec imaging Systems GMBH, Unterschleim, Germany and EchoPAC version 202, GE Vingmed Ultrasound, Horten, Norway). After selection of an appropriate PSAX view, the endo- and epicardium line at end-diastole was automatically generated and manually fine-tuned if tracking accuracy was incorrect. We assessed and recorded left ventricular end-diastolic area (EDA), left ventricular end-systolic area (ESA), fractional area change (FAC), endocardial global circumferential strain (GCS) and global radial strain (GRS). Apical long-axis view with ejection fraction measurement is not feasible in sheep due to peculiarities in their thorax anatomy. Thus we used FAC to describe left ventricular function instead. FAC was defined as the EDA minus the ESA, divided by the EDA. GCS was defined as the strain derived from the deformation of the endocardium in the circumferential direction in the left ventricular short axis. GRS was defined as the strain derived from the whole thickness of the myocardium in the radial direction.


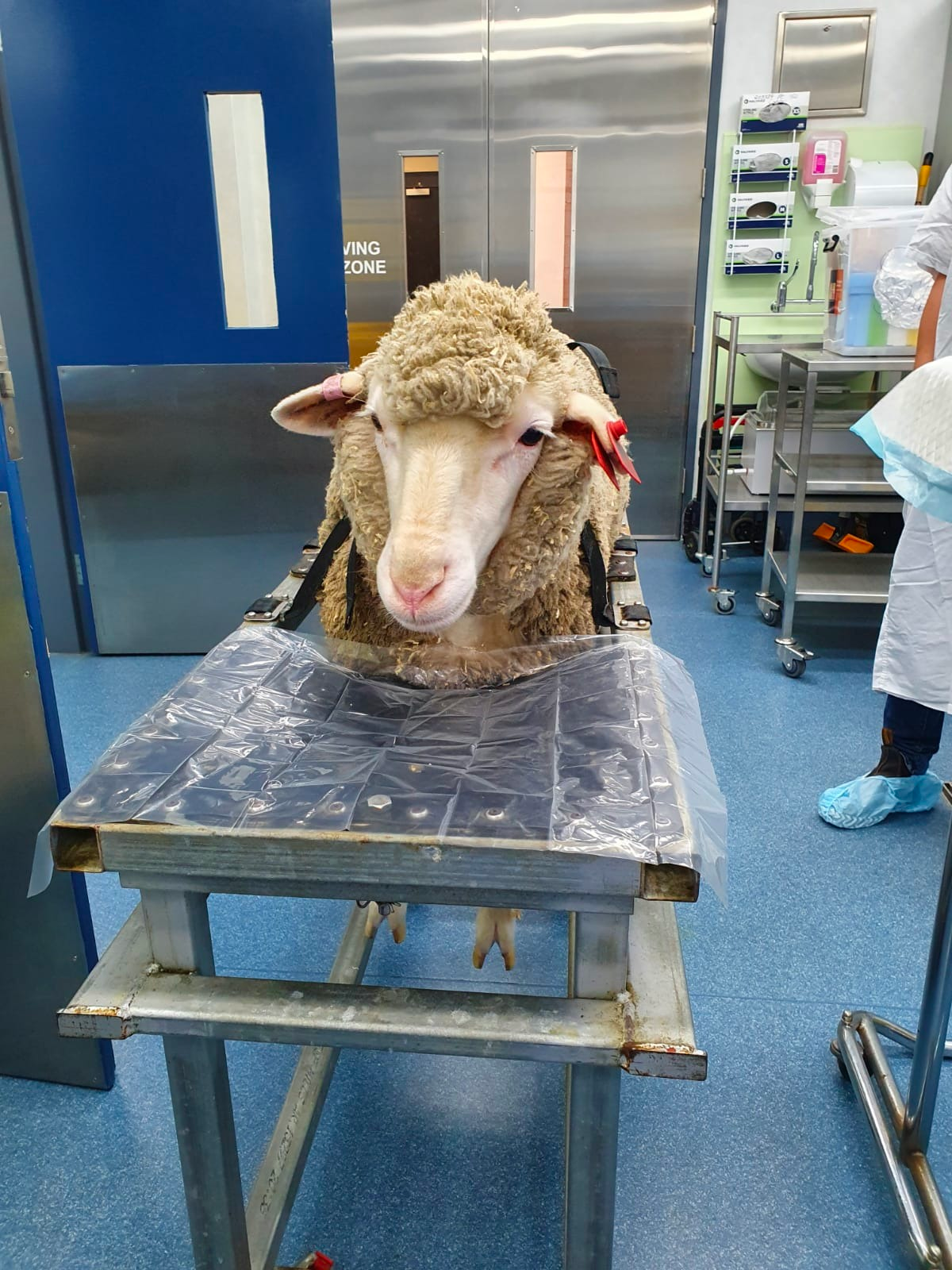


**Figure S1**. Sheep prior to induction and endotracheal intubation restrained into a custom-made trolley.


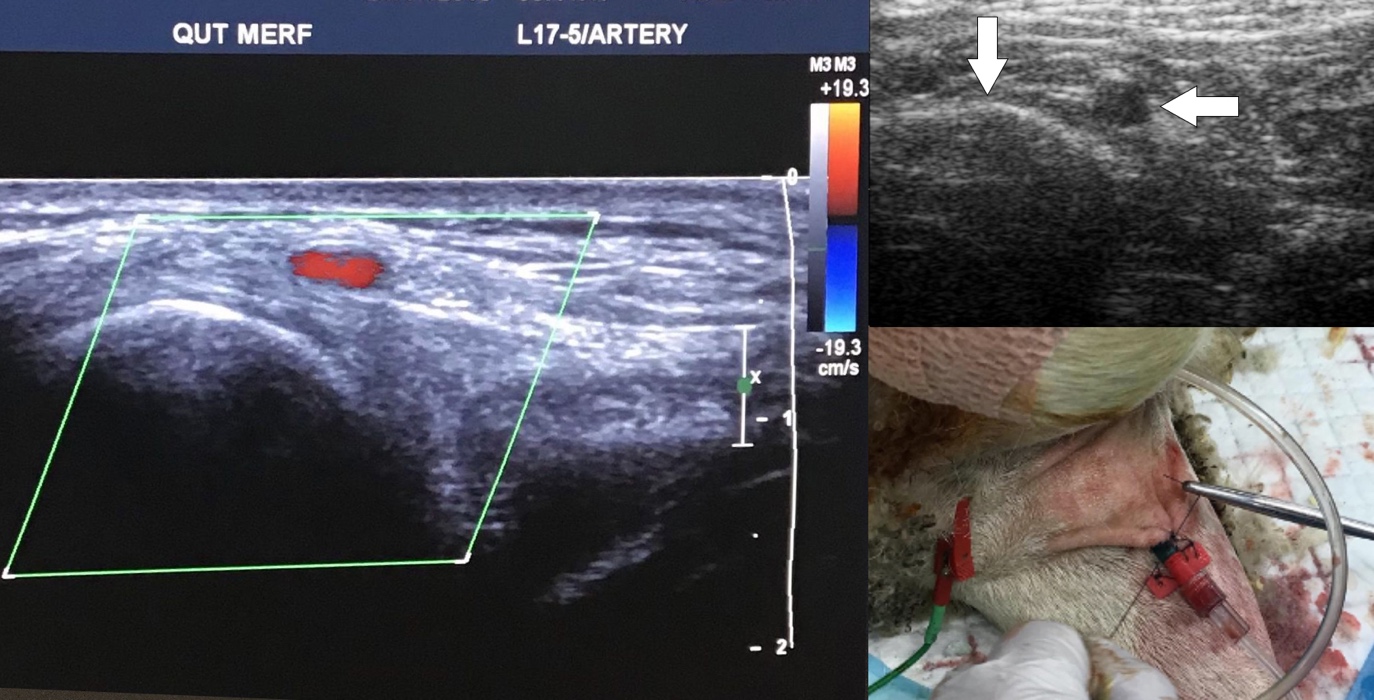


**Figure S2.** **Radial artery cannulation.** Left image shows Doppler ultrasound view of radial artery within the colour box. Top right image shows ultrasound view of the radial artery (right arrow), while the radius is highlighted by the left arrow. Bottom right image depicts how the arterial cannula was secured to the skin.


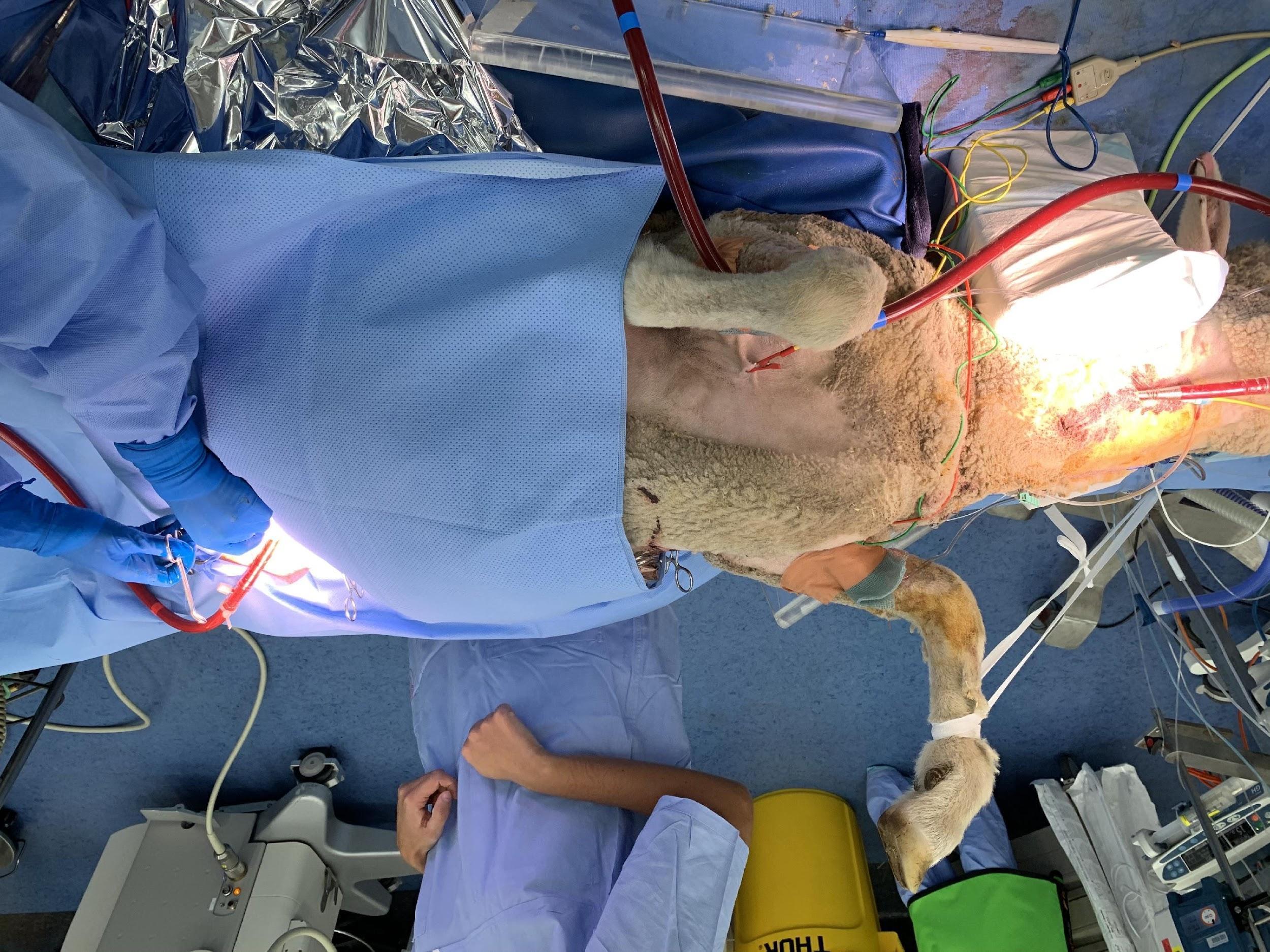


**Figure S3**. Animal in supine position after venous-arterial extracorporeal membrane cannulation through the right jugular vein (inflow) and left femoral artery (outflow).


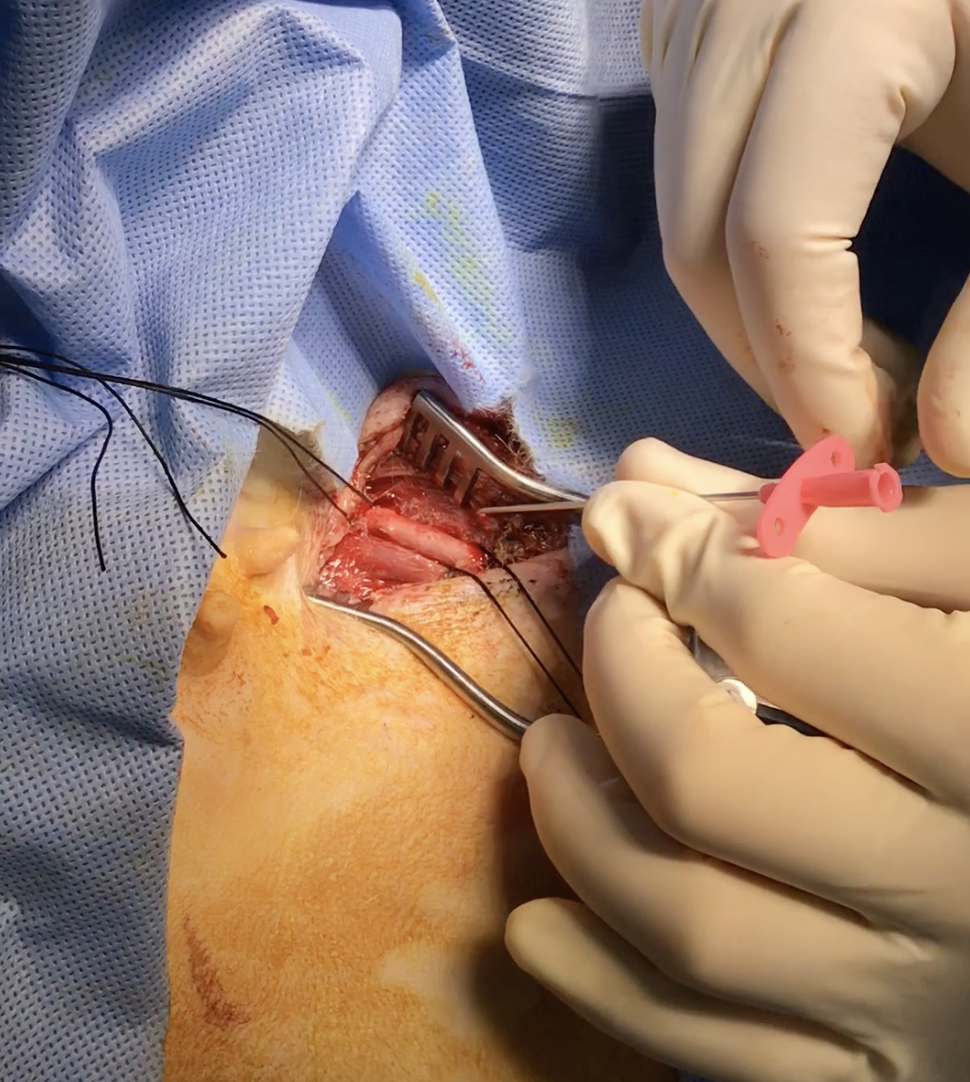


**Figure S4**. Left femoral artery was accessed through cut-down technique prior to cannula insertion.
